# Supplementary figures and images for: Comprehensive single cell analysis of pandemic influenza A virus infection in the human airways uncovers cell-type specific host transcriptional signatures relevant for disease progression and pathogenesis
Source: Front Immunol. 2022 Oct 4;13:978824. doi: 10.3389/fimmu.2022.978824 (PMC9576848; doi:10.3389/fimmu.2022.978824)

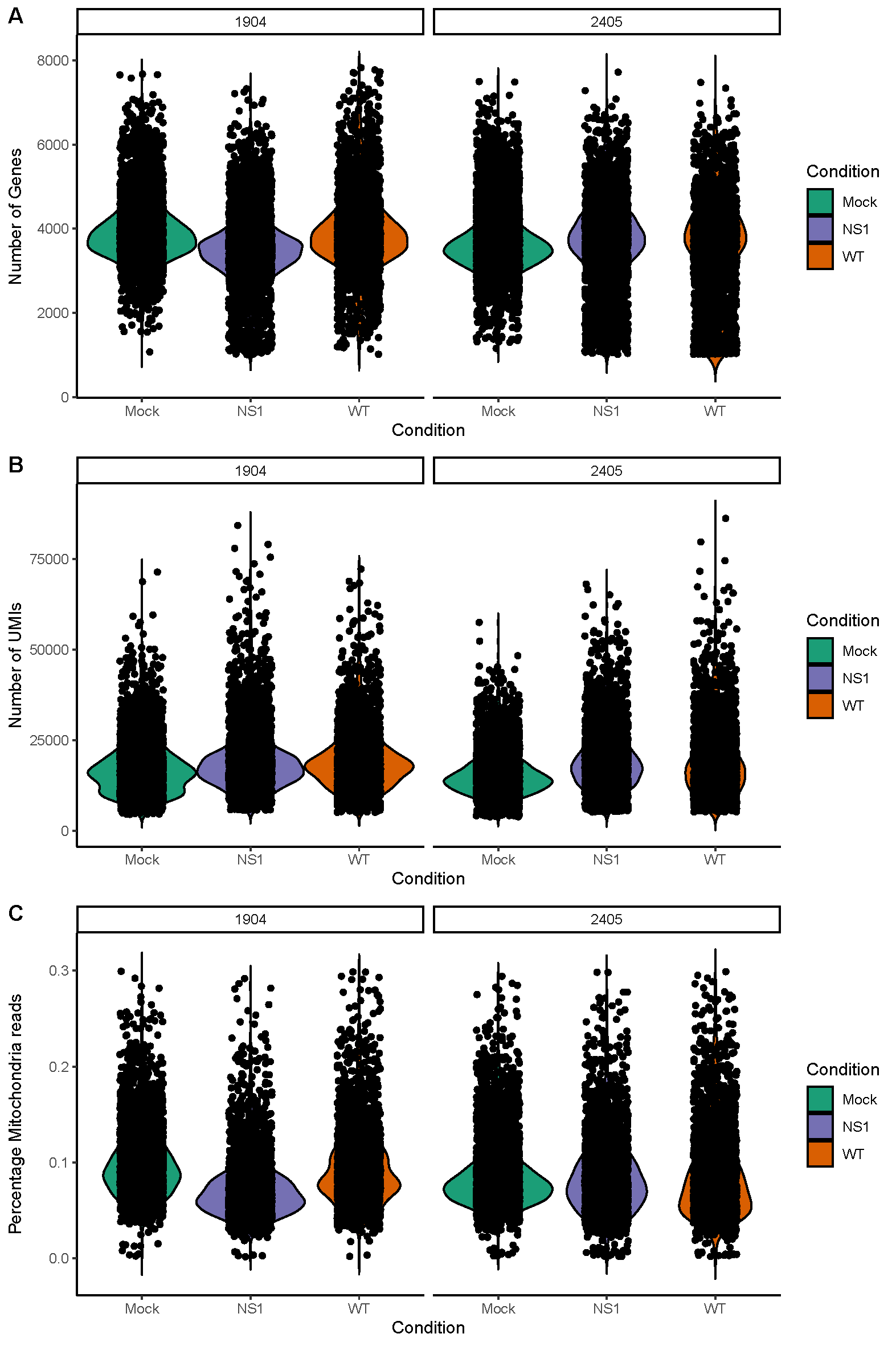

Supplement: Supplementary file 1 [file Image_1.tif]

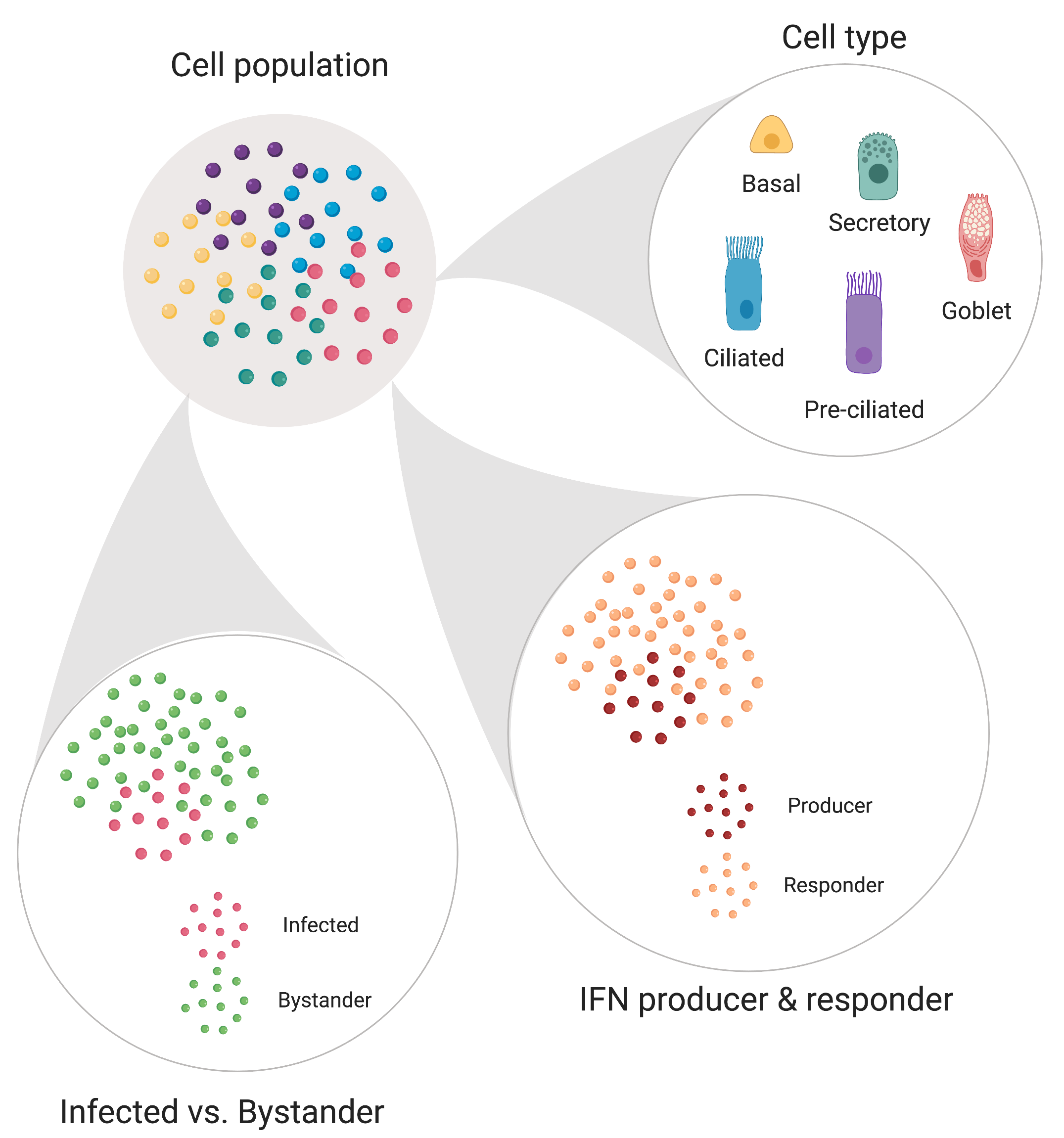

Supplement: Supplementary file 2 [file Image_2.png]

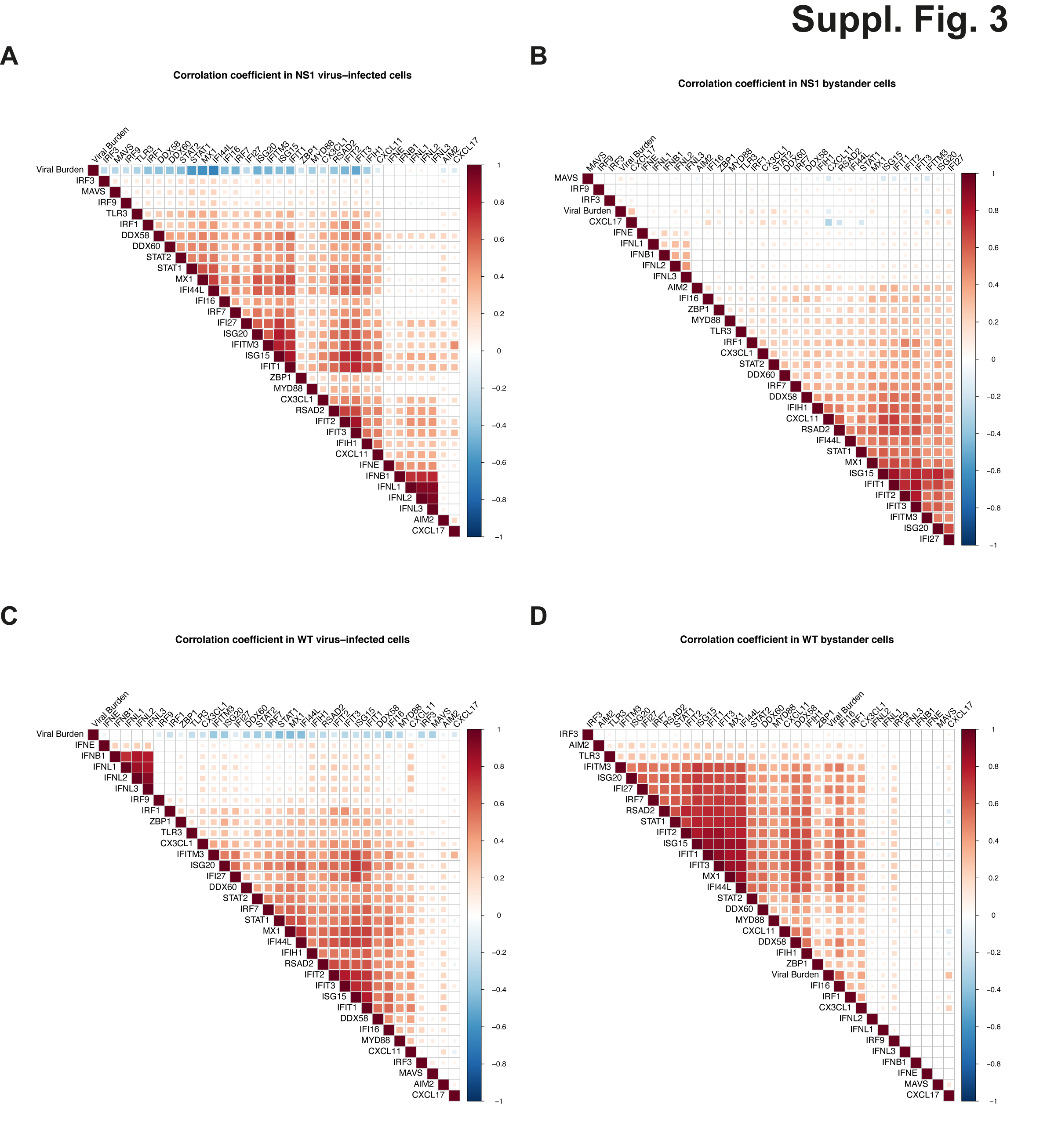

Supplement: Supplementary file 3 [file Image_3.tif]

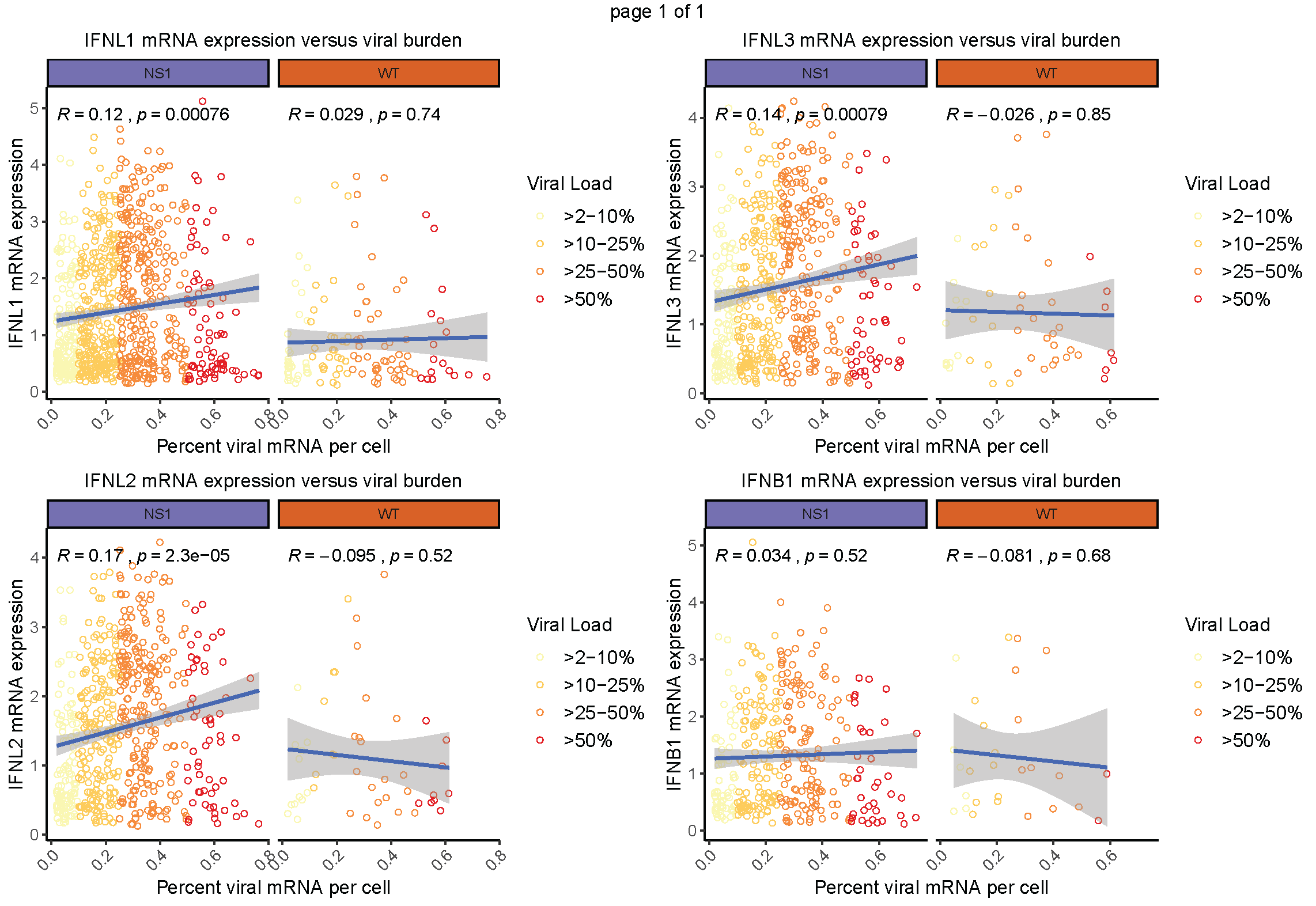

Supplement: Supplementary file 4 [file Image_4.tif]
